# Examining wage inequality among women in India: A multidimensional analysis of socio-economic disparities

**DOI:** 10.1371/journal.pone.0320940

**Published:** 2025-04-24

**Authors:** Anam Pandoh, Ashish Singh

**Affiliations:** Shailesh J. Mehta School of Management, Indian Institute of Technology, Mumbai, India; Imperial College London, UNITED KINGDOM OF GREAT BRITAIN AND NORTHERN IRELAND

## Abstract

Using the nationally representative Indian Human Development Surveys 2004–05 and 2011–12 and multiple inequality measures/frameworks, we investigate both vertical (within-group/interpersonal) and horizontal (between-group/inter group) socioeconomic (based on caste, religion, location and region) inequalities in wages among women in India. We find that the wage inequality (WI) is extremely high (around 60%) and has increased during 2004–12 driven by within-group inequalities which are very high and have increased, whereas between-group inequalities have reduced. There are stark rural-urban divides be it wage labour participation or mean wages; at the same time the WI itself is substantially higher in urban areas. Caste-based WIs are enormous with women belonging to scheduled groups and other backward castes earning substantially lower than their “upper” caste counterparts. The wages of Muslim women are consistently lower than women from other religions. There are vast inter-regional WIs, with the regions of Central and East having lower wages but higher inequalities.

## 1 . Introduction

Nations worldwide have experienced substantial economic growth in the twenty-first century, striving to become powerful economies. However, the unequal distribution of this growth has been a subject of ongoing debate since many decades. The issue gained prominence off late with Thomas Piketty’s (2014) work, “Capital in the 21^st^ Century”, which highlighted a rise in inequality to levels of those before World War I, challenging the foundations of a just society [1]. The dynamics of growth and inequality are complex [2], and numerous studies have tried to decipher this relationship. Adam Smith (1776) advocated for an unequal distribution of income, stating that a larger share of income for capitalists could spur economic expansion. This perspective was followed by several scholars who supported income inequality as a catalyst for economic growth [3,4]. Conversely, other studies have argued that high inequality impedes economic growth as well as the poverty alleviation effects of growth, [5,6] and even if it has positively affected growth, the effect has been temporary [7].

Given the above context, in the developing world, India has achieved remarkable success in terms of economic growth in the last three decades or so, but its growth story is often not considered inclusive [8–12]. Also, in contrast to the literature on decreasing absolute poverty since 1990s, [13] shed light on the wealth concentration among top earners and a sharp, continuous rise in income inequality [14,15].

The inequality discourse in India encompasses two primary dimensions: vertical and horizontal inequalities. Vertical inequalities refer to interpersonal (within-group) differences in economic (such as, income or consumption expenditure) or non-economic (education, health etc.) outcomes, while horizontal inequalities denote disparities (in economic/non-economic outcomes) between groups, based on factors, such as, caste, religion, gender, and other social categories [16–18]. India’s rich diversity in terms of castes, religions, regions, occupations, and economic strata, while culturally enriching, often obfuscates profound socio-economic inequalities [19,20].

In the postcolonial India, socioeconomic advancements have been significant, yet the economic challenges of marginalized groups remain inadequately addressed, perpetuating economic disparities across gender, caste and religious affiliations [21,22]. Although the major economic reforms (of 1991 and thereafter) have improved the economic conditions among these groups, persistence (and increase in) substantial economic inequalities both within and between groups [23], have led to the marginalized sections populating the bottom quintile of the economy [20,24,25]. It is worth noting here that apart from being at the lowest spectrum of economic prosperity and having experienced perpetual discrimination since the historical times, Scheduled Castes (SCs) and Scheduled Tribes (STs) also have high (and increasing) prevalence of within-group inequalities, both in rural and urban areas [26,27].

It is important to take a pause here and note that given the diversity, divide and elements of discrimination against certain sections in the Indian society, most of the discussion on inequality in India has centered around between group differences and the examination of inequality within groups has been limited [26]. Also, the examination of inequalities in economic and non-economic outcomes within women is even more sparse and that too is limited mostly to narratives around the “between” men-women divide in mainly non-economic outcomes, such as, education [28]. That said, gender-based wage inequality in India presents a complex issue, influenced by diverse state-specific traditions, political, and social norms [29]. Despite women constituting half of the population, their workforce participation remains below one-third [30]. [27] shows how economic factors and social identity intersect where women often face disadvantages in wages relative to men, but this disparity is compounded when considering their social group identity. Thus, there are a few studies which have examined the gender based (between men-women) wage gap in India [31] and a few [32] which have estimated inequality of opportunities in income and consumption expenditure (both per capita basis), we (to the best of our search) could not find any study which has comprehensively examined wage inequalities within women in the context of India. We find this research gap as critical, especially considering evidence of substantial and varying within group disparities at different levels in India [26,33].

This study therefore aims to fill the aforesaid research gap by examining wage disparities among women across various social groups in India, thus analysing the association of social backgrounds with economic outcomes in the diverse and multifaceted Indian society.

## 2. Data and estimation

### Description of data

We use data from the two rounds of the Indian Human Development Survey (IHDS) conducted in 2004–05 (IHDS - I) and 2011–12 (IHDS – II), respectively. The IHDS, a comprehensive nationally representative multi topic survey, is conducted by the National Council of Applied Economic Research, New Delhi, in collaboration with the University of Maryland, covering all states and Unition Territories of India (except the Andaman/Nicobar and Lakshadweep islands) [34]. IHDS dataset is chosen over other large, frequent surveys like National Sample Survey Office (NSSO) Employment-Unemployment Survey (EUS) due to its longitudinal nature and rich socioeconomic data. While NSSO EUS is cross-sectional and provides national-level employment trends for different individuals in each round, IHDS tracks the same households over two waves (2004–05 and 2011–12). This panel structure enables an analysis of wage mobility and long-term inequality trends, which is central to our research objective. Moreover, the IHDS collects detailed (household and individual level) information on economic (such as, income and wages) as well as non-economic indicators, such as education and health; it includes various modules encompassing economic status, education, employment, fertility, gender relations, health, marriage, nutritional status etc. [34]. In 2004–05, 41554 households (26734 rural and 14820 urban) were covered whereas the 2011–12 round included 42152 households (27580 rural and 14573 urban).

This study focuses on women across all the states of India, into six regions, namely, North (Jammu & Kashmir, Himachal Pradesh, Delhi, Uttaranchal, Punjab, Haryana and Rajasthan), Central (Uttar Pradesh, Madhya Pradesh and Chhattisgarh), East (Bihar, Jharkhand, West Bengal and Orissa), North-east (Assam, Arunachal Pradesh, Meghalaya, Manipur, Tripura, Nagaland and Sikkim), West (Maharashtra, Goa and Gujarat), and South (Andhra Pradesh, Karnataka, Kerala, Tamil Nadu and Pondicherry) [32]; (kindly see [20] for a detailed discussion on the categorization of states into regions). This categorisation also as per the grouping followed by the Ministry of Health and Family Welfare, Government of India which collects and monitors health and other family welfare related indicators (through the National Family Health Surveys) in India.

The study specifically targets women who were 15 years or older, were not enrolled in school or college at the time of survey but were on wages (both casual or permanent in nature), and were not receiving income from other sources, such as, farming or business ventures. These women were then categorised based on social factors, like caste, religion and location of residence (rural/urban). For analytical purposes, (following [32], pp. 395) caste has been divided into three categories; “Other Castes (OC)” [also “General” or “Upper Castes”], “Other Backward Castes” (OBC) and “Scheduled Castes and Scheduled Tribes” (SC/ST); women belonging to SC/ST community have experienced exclusion (physical and social) and discrimination since historical times and lag behind the non-Scheduled groups in various indicators of welfare [19]. Similarly, religion has been grouped into the three categories of “Hindu” (majority in India), “Muslim” and “Others”; Muslims in India lag their Hindu counterparts to a large extent in various outcomes including education, income and employment [32,35].

### Methods – measures of inequality used in the analysis

We used the Gini Index mainly for within group (interpersonal/vertical) inequalities (for example, inequality in wages within the SC women) due to the following reason: GI is a summary indicator for measuring inequality in a society or group (varies from ‘0’ in case of perfect equality to ‘1’ [or 100 in percentage terms] in case of perfect inequality [only one individual has all the incomes and all the rest have zero income]) and satisfies the four basic axioms/properties desirable in an inequality measure—i.e., (a) Anonymity principle; (b) Population Replication/Invariance Principle; (c) Scale Invariance/Mean Independence/Relative Income Principle; and (d) Dalton Transfer Principle (for details, please refer to [36], pp. 174–184; [37]; [38], pp.1324). The Gini index can be written as:


$$G\;=\;\frac{1}{2n^{2}\mu }\sum_{i=1}^{n}\sum_{j=1}^{n}\left|w_{i}-w_{j}\right|$$

where G is the Gini index, *i* and *j* are any two individuals of the group, *n* denotes the number of women in the group, W*i* and W*j* are the wages of *i*-th and *j*-th woman, respectively and µ  is the mean wage of the group. The wage figures used in this study are nominal wages and have not been adjusted for inflation. Since we employ the Gini coefficient to measure within-group wage inequality, the use of nominal or real wages does not impact our findings. The Gini coefficient satisfies the property of scale invariance, meaning that multiplying all wages by a constant factor (such as a price index) does not change its value.

Gini coefficient provides an initial overview of wage (interpersonal) inequality among women across various dimensions such as geographical regions, castes, religions, and locations. To delve deeper into the nature of these disparities, and to decompose the overall inequality into within group and between group (for example, if groups are based on caste, then within caste and between caste) components; we used Mean Log Deviation (MLD) as the inequality measure. We used MLD (also known as GE (0)) to decompose the inequality in wages into within type and between type components because it is the only measure of inequality which satisfies the four basic axioms (anonymity or symmetry; population replication or replication invariance; mean independence or scale invariance; and Pigou–Dalton principle of transfers) described above as well as the two additional axioms/properties of additive subgroup decomposability (ASD) and path independence (PI) [20,37]. MLD is chosen over other measures of the generalized entropy class like the Theil’s index as they satisfy five of the axioms mentioned but fail to satisfy the path independence property therefore making them less desirable for the present study. The additional properties of ASD and PI are particularly important for our study; the ASD is important because the study primarily decomposes the total wage inequality into within-group and between-group components; and the property of PI is also required in the sense that the decomposition must yield the same result or the decomposition should be invariant to whether within-group inequality is eliminated first and the between-group component computed second, or the reverse [20,37]. The Mean Log Deviation decomposition formula is:


$$MLD_{overall}\;=\;MLD_{within\;group}\;+\;MLD_{between\;group}$$

$$MLD=\sum_{g=1}^{G}\frac{n_{g}}{n}MLD_{g}+\sum_{g=1}^{G}\frac{n_{g}}{n}\ln\left(\frac{\overset{―}{y}}{\overset{―}{y_{g}}}\right)$$

Where G is the number of groups (caste groups, religious groups, location groups), n_*g*_ is the population of group *g*, n is the total population, MLD_*g*_ is Mean Log Deviation within group *g*, ȳ_*g*_ is the mean wage of group *g* and ȳ is the overall mean wage.

Women have been categorized into different groups based on caste, religion, location (rural/urban) and geographical region; and the decomposition of overall inequality into within group and between group components has been carried out for each categorization separately. This categorization allows for a nuanced analysis of wage inequality, breaking it down into between-group and within-group components. For instance, as mentioned earlier, the categorization of women based on caste results in three sub-groups – “Others”, “OBCs” and “SC/ST” women population. If we now decompose the overall inequality in wages into within and between components using MLD, then the between-group component is nothing but the caste-based inter group (horizontal) inequality/disparity, whereas, the within-group component will give us the weighted sum of inequalities within the Others, OBCs and SC/STs. A similar analytical approach is applied to other categorizations, such as, religion and location, at both the regional and national levels.

## 3 . Results

### 3.1 Trends in women on wages and their mean annual wages in India

Table 1 depicts percentage distribution of women on wages across different socio-demographic factors in IHDS I (2004–05 and IHDS II (2011–12). Proportion of women wage earners is higher in rural areas than urban areas, both in 2004–05 and 2011–12. Figures in the table indicate percentage distribution of women on wages between rural and urban settings. However, for a more nuanced comparison, we have calculated percentage of women on wages for rural and urban areas separately as well*:* 23.3% in rural areas and 13.2% in urban areas (2004–05) and 27% in rural and 16.2% in urban areas (2011–12). These figures remain consistent with the overall pattern in PLFS report, by NSSO, MoSPI (2023–24) [39]. Nationally and in almost all the regions (barring South) Scheduled Caste/Tribe (SC/ST) women consistently have the highest share among the women wage earners, accounting for about 46% in 2004–05 and 44% in 2011–12. In the region of South, it is the women belonging to the Other Backward Castes (OBC) who have the highest share in both 2004–05 and 2011–12. The possible reason for the above two observation lies in the fact that women belonging to SC/ST followed by OBC contribute majorly to the women in (on) casual jobs (labour); and the proportion of women in casual jobs (labour) is very high in the total number of women on wages [33,40]; also, the households belonging to “Other” (Upper) castes generally don’t allow the women of their households to work on casual jobs (or casual labour) [33,41,42].

**Table 1:** Percentage distribution of women on wages by social groups in India: 2004-05 & 2011-12.

Socioeconomic Characteristics	2004-05							2011-12						
	North	Central	East	NE	West	South	All India	North	Central	East	NE	West	South	All India
***Caste***														
Others	32.32	12.28	21.66	25.51	23.77	12.03	17.18	28.46	26.95	23.73	25.51	22.28	11.85	18.03
OBC	25.24	33.96	24.74	16.21	34.72	45.53	36.94	25.88	10.91	24.58	12	35.02	48.8	38.31
SC/ST	42.44	53.76	53.6	58.28	41.51	42.44	45.89	45.51	72.97	51.69	62.49	42.7	39.35	43.66
***Religion***														
Hindu	80.5	89.8	81.8	44.55	84.12	89.9	86.36	76	84.09	81	55	83	88	83
Muslim	10.81	9.16	12.58	12.62	5.48	5.37	7.54	11.73	15	15	8	5	7	10
Others	8.68	1.04	5.62	42.82	10.41	4.73	6.11	12.48	1	4	38	12	5	6.9
***Location***														
Rural	57.73	77.39	84.64	62.4	68.31	76.98	75.17	52.86	69.63	80.31	65.82	60.28	64.16	65.92
Urban	42.27	22.61	15.36	37.6	31.69	23.02	24.83	47.14	30.37	19.69	34.18	39.72	35.84	34.08
***Region***														
North	–	–	–	–	–	–	6.53	–	–	–	–	–	–	10.19
East	–	–	–	–	–	–	14.89	–	–	–	–	–	–	13.84
Central	–	–	–	–	–	–	15.86	–	–	–	–	–	–	17.56
NE	–	–	–	–	–	–	1.41	–	–	–	–	–	–	2.56
West	–	–	–	–	–	–	17.58	–	–	–	–	–	–	15.75
South	–	–	–	–	–	–	43.74	–	–	–	–	–	–	40.1

Source: Authors’ calculations based on IHDS data 2004–05 and 2011–12.

In terms of religious groups, Hindu women contribute substantially higher share to women on wages nationally (as well as in all the regions) across both time periods compared to women of other religious groups. Meanwhile, the share of Muslim women (which is quite low) increased modestly from 7.5% to 10% during the study period. Regionally, the North-East region presented a distinct picture in terms of religious demographics. Contrary to the other regions, where Hindu women constituted 80–90% of the share of the women on wages, the North-East had women belonging to “Other” religious groups contributing 43% and 38% to the women on wages in 2004–05 and 2011–12, respectively.

The mean annual wages (henceforth mean wages should be read as mean annual wages) for the Indian women across social groups and regions are presented in Table 2. Women in urban areas have significantly higher wages, with more than triple of the wages in rural areas both in 2004–05 and 2011–12. This urban-rural wage gap underscores the persistent economic challenges for rural women wage earners. Central region followed by the Eastern region have the lowest mean wages in India, while the North-East has the highest, both in rural and urban settings. At the national as well as regional level, caste-based wage disparities are profound with SC/ST women consistently earning the least, followed by the OBC women. This is not surprising, because this trend is observed in almost all desirable demographic-economic and social outcomes in India. Among all the regions, the SC/ST and OBC women earn the least in the Central region. In the religious groups, women of “Other” religions consistently earned highest mean wages, both nationally as well as in different regions. Muslim women, on the other hand, consistently earned the least. The North-East continued to provide better wage opportunities for the women belonging to Hindu and “Other” religious groups during the study period. The regions of Central and East, however, presented less favourable wage conditions for Hindu and Muslim women. The Kernel density graphs (presented in) [Supporting information] indicate increased wage variation or inequality in 2011–12 compared to 2004–05. Also, urban women not only had higher average wages but also exhibited greater wage dispersion compared to their rural counterparts.

**Table 2:** Mean Annual Wages of women across social groups in India: 2004-05 & 2011-12.

	2004-05							2011-12						
Social Groups	North	Central	East	NE	West	South	All India	North	Central	East	NE	West	South	All India
***Caste***														
Others	55,529	24,218	29,816	36,201	25,676	26,732	32,962	1,14,119	62,381	56,667	1,10,560	54,426	56,150	75,114
OBC	23,014	7,191	14,251	66,491	13,420	12,083	13,032	38,468	17,314	27,725	1,04,067	27,004	34,105	35,501
SC/ST	14,782	6,199	11,002	54,802	10,985	9,425	11,773	33,486	16,204	22,721	1,12,983	26,980	25,460	30,184
***Religion***														
Hindu	31,432	8,155	16,481	46,393	15,832	12,363	15,046	62,402	21,888	32,432	1,05,053	33,256	32,441	37,466
Muslim	27,166	12,742	7,717	20,772	10,278	11,958	13,855	39,605	21,939	14,853	34,634	28,300	29,348	27,580
Others	43,125	29,742	25,383	62,598	16,678	27,883	34,792	46,592	2,96,550	58,400	1,29,116	34,448	51,574	67,640
***Location***														
Rural	15,562	5,202	7,651	33,499	8,035	8,958	8,972	27,135	12,096	15,937	60,795	20,598	23,186	21,999
Urban	48,127	20,682	33,990	72,703	33,993	22,491	31,921	94,802	49,249	62,174	1,76,135	61,291	49,730	68,162
***Total***	32,059	8,914	16,148	52,859	15,570	13,102	16,360	56,398	23,636	31,731	1,11,079	33,025	33,290	39,049

Source: Authors’ calculations based on IHDS data 2004–05 and 2011–12.

### 3.2 Further evidence on distribution of wages across socioeconomic characteristics

**Fig 1:**
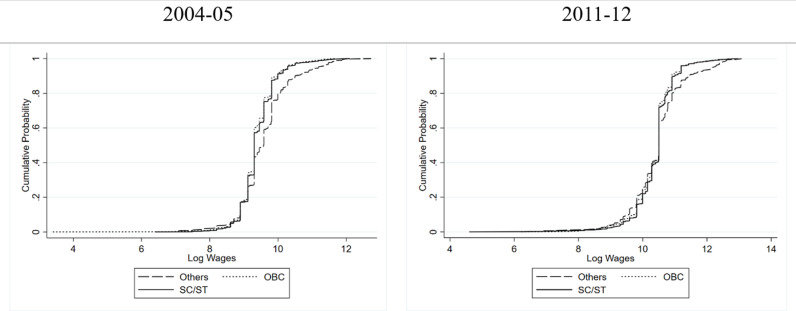
Cumulative Probability Distribution of Wages of Women in Rural India conditional on Caste.

**Fig 2:**
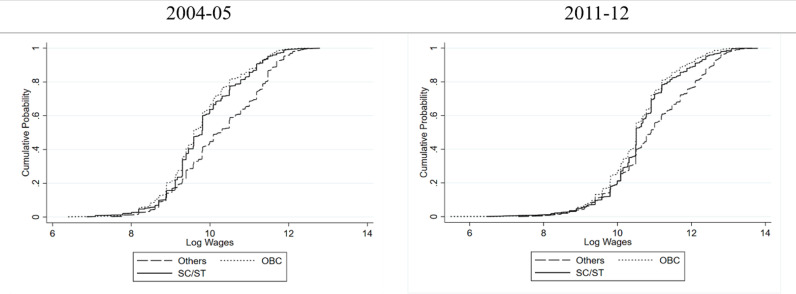
Cumulative Probability Distribution of Wages of Women in Urban India conditional on Caste.

Figs 1 and 2 show the persistent skewness in wage distribution (conditional on caste) for the two periods, 2004–05 and 2011–12; the 2004–05 figure for rural areas shows a consistent wage disadvantage for SC/ST women, with their Cumulative Distribution Function (CDF) remaining towards the left of OBCs and Others, indicating a persistent low-wage bias. However, the 2011–12 data reveals a nuanced shift: the SC/ST women wage distribution curve suggests some early rise towards higher wages but at a lower probability level, hinting at early occupational entry or limited upward mobility, and thus a concentration in low-wage sectors. Notably, in almost over the whole range (especially at the higher wage levels) the curve of wages for women of “Other” caste group remains above the curves of wages for SC/ST and OBC women, indicating that despite some gains, disadvantaged groups are less represented at the medium to higher wage levels, possibly due to enduring structural barriers, discriminatory practices, or limited access to high-paying occupation. In the urban landscape of 2004–05, wage distribution shows a higher concentration of women in the lower wage brackets, shifting towards a more balanced distribution in 2011–12.

Furthermore, caste-based wage gap was more pronounced in 2004–05, particularly around the 60th percentile (cumulative probability of 0.6), where the log wage gap between SC/ST and ‘Others’ was wider. Over time, this gap narrowed, especially at lower wage levels (up to the 40th percentile), suggesting that SC/ST women experienced greater wage gains at the bottom of the distribution. However, disparities remain significant at higher wage levels. Comparing these urban findings with rural counterparts unveils intriguing disparities. The caste wage gap, while persistent in both settings, is relatively higher in urban areas. This divergence could be attributed to factors, such as, in the absence of high paying jobs (and most of the jobs being casual in nature) in rural areas, the disparity in wages is relatively lower compared to urban areas (similar argument can be found in [20]).

**Fig 3:**
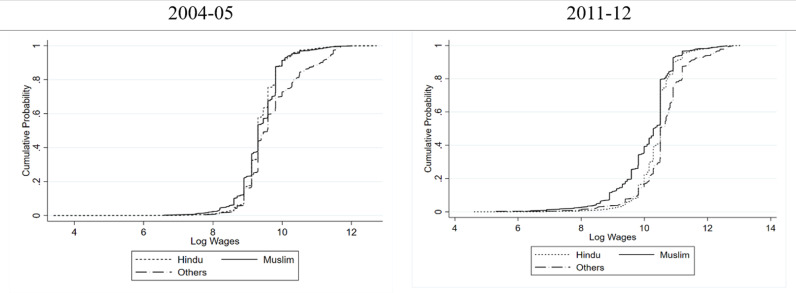
Cumulative Probability Distribution of Wages of Women in Rural India Conditional on Religion.

**Fig 4:**
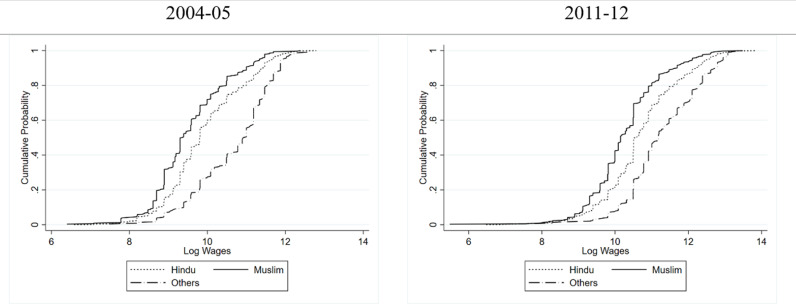
Cumulative Probability Distribution of Wages of Women in Urban India Conditional on Religion.

Figs 3 and 4 show a consistent wage disparity among the women of three religious groups. A larger concentration of women at the lower end of the wage spectrum is evident across all religious groups. In the rural areas, almost throughout the distribution, the women belonging to the “Others” religious group are earning more than their Hindu and Muslim counterparts in both 2004–05 as well as 2011–12. Between Hindu and Muslim women, it is the Hindu women who are earning more wages over most of the distribution. In urban settings, the wage gap is more pronounced and clearer. In both the years, women belonging to “Others” religious group earn higher wages and Hindu women who in turn earn higher wages than the Muslim women in the urban areas as well. Also, urban women from all religious groups exhibited higher median wages than their rural counterparts, suggesting an urban wage premium for women in India. Moreover, as in the case of caste, religion-based inequality in wages is higher in urban areas compared to rural ones.

**Fig 5:**
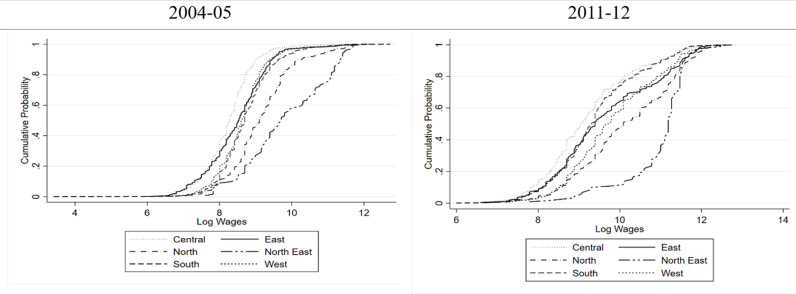
Cumulative Probability Distribution of Wages of Women in Rural India Conditional on Geographical Region.

**Fig 6:**
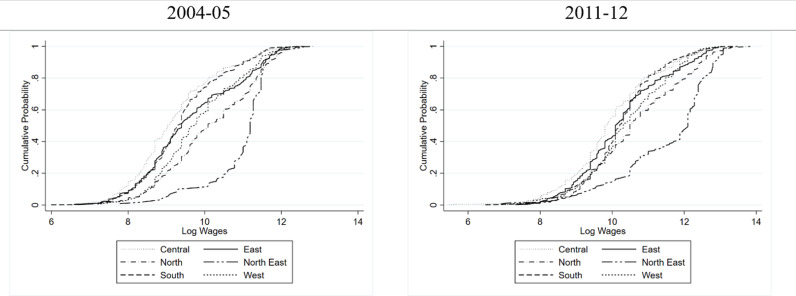
Cumulative Probability Distribution of Wages of Women in Urban India Conditional on Geographical Region.

Figs 5 and 6 reinforce the observation that women in the North East region earn consistently higher wages over almost whole of the distribution, whereas, those in the Central region earned the lowest over almost whole of the distribution. Also, the region-based disparities in wages look to be narrowing in the rural areas, Further, the region-based variations in wages are more in urban areas compared to the rural areas.

### 3.3 Inequality estimation using Gini and MLD

Table 3 presents the Gini estimates (of inequality) for the annual wages among Indian women across different social and geographical backgrounds for the years 2004–05 and 2011–12. It can be observed that the overall wage inequality among women is extremely high (around 60%) and has increased from 0.59 in 2004–05 to 0.62 in 2011–12. Regionally, the Eastern region exhibited the highest inequality in 2004–05 (0.62), whereas the Southern region had the lowest (0.52). In 2011–12, the North, Central and Eastern region showed the highest wage inequality (0.67, 0.67 and 0.65 respectively), with the Southern region again recording the lowest (0.54). As mentioned earlier, the regions of East and Central which have high inequalities in 2004–05 and 2011–12, respectively comprise of the poorest states of India.

**Table 3:** Wage Inequality (based on Gini coefficient) among women in India (and regions): 2004-05 and 2011-12.

	2004-05							2011-12						
Social Groups	North	Central	East	North East	West	South	All India	North	Central	East	North East	West	South	All India
*Caste*														
Others	0.53	0.65	0.62	0.53	0.63	0.61	0.62	0.59	0.65	0.65	0.59	0.55	0.61	0.63
SC/ST	0.50	0.44	0.54	0.47	0.52	0.44	0.58	0.61	0.62	0.52	0.63	0.52	0.44	0.59
OBC	0.55	0.53	0.56	0.46	0.59	0.49	0.57	0.65	0.60	0.63	0.67	0.52	0.55	0.60
*Religion*														
Hindu	0.62	0.56	0.60	0.56	0.61	0.51	0.61	0.67	0.66	0.62	0.65	0.56	0.54	0.62
Muslim	0.56	0.61	0.38	0.53	0.53	0.51	0.58	0.68	0.63	0.37	0.40	0.52	0.52	0.58
Others	0.54	0.64	0.65	0.34	0.63	0.56	0.59	0.64	0.18	0.59	0.56	0.57	0.55	0.63
*Location*														
Rural	0.52	0.41	0.5	0.52	0.39	0.43	0.47	0.58	0.55	0.50	0.65	0.36	0.48	0.52
Urban	0.53	0.61	0.61	0.33	0.55	0.57	0.59	0.61	0.63	0.62	0.45	0.54	0.54	0.62
*Total*	0.61	0.58	0.62	0.53	0.61	0.52	0.59	0.67	0.67	0.65	0.63	0.56	0.54	0.62

Source: Authors’ calculations based on IHDS data 2004–05 and 2011–12.

Urban areas have substantially higher wage inequality than the rural areas both in 2004–05 and 2011–12, except in the North East. Also, the wage inequality increased in both rural as well as urban areas during 2004 to 2012 period. In case of religion, the wage inequality has increased slightly among women belonging to Hindu and “Other” religions whereas it has remained same among the Muslim women. In 2004–05 the wage inequality among the Hindu women was slightly higher than the women belonging to the “Other” religions but in 2011–12 this got reversed. Caste-wise, in 2004–05, the lowest wage disparity was among the OBC women (0.57) with the wage disparity among SC/ST women (0.58) almost being same as that of the OBC women and highest among “Others” category. This trend got changed in 2011–12 where the wage inequality among the OBC and SC/ST women became 0.60 and 0.59, respectively. Also, the wage inequality increased for all the caste categories.

Gini coefficient provides an initial overview of wage inequality (within group) among women belonging to different groups, such as, those belonging to – different castes; different religions; rural/urban; and different geographic regions. For example, the wage inequality among the Muslim women (or within the Muslim women) was 0.58 both in 2004–05 as well as 2011–12. But to get a complete picture, we also need to get the estimates of between group wage inequalities, such as, wage inequality between – different caste groups; religious groups; rural/urban; and different geographical regions. For example, if we need to get the estimates of wage inequality between the women of different religions (Hindu, Muslim and Others); we need an inequality measure which can decompose the overall inequality into the within group and between group components. That is, the inequality measure should decompose the total inequality among the women into the within (Hindu, Muslims and Others) religion and between (Hindu, Muslim and Others) religion components. While, between-group component is nothing but religion-based inequality, within-group component is the wage disparity within the sub-groups (Hindu, Muslim and Others). It may also be helpful to note that the within component is the weighted sum of the inequality within each subgroup. For example, the within component in case of religion-based decomposition is the weighted sum of within inequality in – Hindus, Muslims and Others. As mentioned in the “Data and Methods” section, MLD is the measure used for decomposition for reasons already discussed. This decomposition (analytical exercise) has been applied for all the categorizations, such as those based on – caste; religion; location (rural/urban); and geographical regions. Further, the decomposition based on caste, religion and location has been carried out at national as well as regional level.

The insights from the decomposition of total inequalities into the within and between components for various groups using MLD for 2004–05 and 2011–12 are presented in Table 4. The first observation is that the results for overall wage inequalities corroborate the findings using Gini coefficient; for example, the overall wage inequality among women increased during the 2004–2012 period; the lowest regional inequality was in the South in 2004–05 as well as 2011–12. Both, Gini and MLD analyses indicate improvements in wage equality in the Western region during 2004–05 to –2011–12.

**Table 4:** Estimates of wage inequality decomposition (within-group and between-group) using Mean Log Deviation, India: 2004-05 and 2011-12.

	2004-05			2011-12		
	Within (% of Total)	Between (% of total)	Total	Within (% of total)	Between (% of total)	Total
***All-India***						
*Caste*	0.54 (85.7)	0.08 (14.3)	0.63	0.64 (90.1)	0.07 (9.9)	0.71
Religion	0.61 (96.8)	0.02 (3.2)	0.63	0.69 (97.1)	0.01 (2.9)	0.71
Location	0.45 (71.4)	0.17 (28.6)	0.63	0.56 (78)	0.15 (22)	0.71
Region	0.57 (90.4)	0.05 (9.6)	0.63	0.66 (92.9)	0.05 (7.0)	0.71
***North***						
Caste	0.52 (71.2)	0.20 (28.8)	0.73	0.77 (82.2)	0.16 (17.8)	0.94
Religion	0.72 (98.5)	0.01 (3.1)	0.73	0.93 (98.8)	0.13 (1.2)	0.94
Location	0.53 (78)	0.19 (22)	0.73	0.73 (77.6)	0.20 (22.3)	0.94
***Central***						
Caste	0.46 (77.9)	0.13 (22.1)	0.59	0.72 (81.8)	0.16 (18.2)	0.88
Religion	0.56 (94.9)	0.02 (5.1)	0.59	0.82 (93.1)	0.06 (6.9)	0.88
Location	0.38 (64.4)	0.21 (35.6)	0.59	0.62 (70.4)	0.25 (29.6)	0.88
***East***						
Caste	0.59 (89.3)	0.07 (10.7)	0.66	0.62 (91.1)	0.05 (8.9)	0.68
Religion	0.63 (95.4)	0.03 (4.6)	0.66	0.64 (94.1)	0.03 (5.9)	0.68
Location	0.50 (75.7)	0.16 (24.2)	0.66	0.51 (75.1)	0.16 (24.9)	0.68
***North East***						
Caste	0.53 (94.6)	0.03 (5.4)	0.56	0.95 (99.5)	0.00 (0.5)	0.95
Religion	0.51 (91)	0.05 (9)	0.56	0.89 (93.6)	0.06 (6.4)	0.95
Location	0.45 (80.3)	0.11 (19.7)	0.56	0.77 (81)	0.17 (19)	0.95
***West***						
Caste	0.60 (89.5)	0.07 (10.5)	0.67	0.51 (87.9)	0.06 (12.9)	0.58
Religion	0.67 (99.5)	0.00 (0.5)	0.67	0.58 (99.9)	0.00 0.1	0.58
Location	0.37 (55.2)	0.30 (44.7)	0.67	0.38 (73.5)	0.19 (26.5)	0.58
***South***						
Caste	0.41 (87.2)	0.05 (13.8)	0.47	0.48 (92.3)	0.03 (7.7)	0.52
Religion	0.45 (95.7)	0.02 (3.8)	0.47	0.52 (99.7)	0.00 (0.3)	0.52
Location	0.38 (80.8)	0.08 (17.2)	0.47	0.46 (88.4)	0.06 (11.6)	0.52

Source: Authors’ calculations based on IHDS data 2004–05 and 2011–12.

It can be observed from Table 4 that the major part of total wage inequality in all the cases is contributed by the within component which in some cases is more than 90% of the total wage inequality. At the all-India level, the between group wage inequality is highest (more than 20% of the total in 2004–05 as well as 2011–12) in the case of rural/urban divide followed by caste-based categorization. In both the cases (and even in the case of religion-based categorization), the between group wage inequality has reduced during the study period. Clearly the increase in total wage inequality during the study period is driven by the increase in within group inequalities.

Also, the between group (region) inequality is high but has reduced during the study period. If we observe the patterns of between group inequalities based on rural/urban, caste and religion in the various geographical regions of India, we find that the between group components are consistently relatively higher in the Central, Eastern and Northern regions. These regions have the poorest states in terms of socio-economic-demographic development. In 2011–12, the rural/urban divide is highest in the Western region where the between component is almost 30% of the total inequality. Also, the caste-based divide is highest in the Northern and Central region (almost 18% of the total).

In summary, the decomposition of wage inequality reveals some very important and interesting patterns of wage disparity among the Indian women, with notable differences across location (rural/urban), regions, castes, and religious groups, and a shift towards greater within-group inequalities during the study period.

## Conclusion and discussion

Almost all of the studies on economic inequality in the Indian context are either based on per-capita (household) consumption expenditure or focused exclusively on men when the economic outcome is captured in terms of wages etc.; and second, a few studies which are there, based on gender and wages are mostly focussed on gender wage disparity/gap between men and women [21,29–31]. In the important debate about the inclusiveness of the Indian economic growth process, one piece is missing and that is, what is the extent of and how wage inequalities within the Indian women have changed over time. This study is perhaps the first to comprehensively and critically examine and analyse the socio-economic inequalities in wages among (or within) women in India. It uses nationally representative data from two rounds of IHDS and therefore investigates the patterns as well as trends in the above-mentioned inequalities.

Some of the main findings of this study are as follows: the women wage earners (casual and regular combined) are predominantly based in rural areas. Nationally and in almost all the regions (except South) Scheduled Caste/Tribe (SC/ST) women consistently have the highest share among the women wage earners, whereas, in the Southern region, it is the women belonging to the Other Backward Castes (OBC) who have the highest share. This is not surprising because of the fact that women belonging to SC/ST followed by OBC communities contribute majorly to the women in casual jobs (or labour) and the percentage share of women in casual jobs (labour) is very high in the total number of women on wages [33,40]; whereas, the households belonging to “Other” (Upper/historically socio-economically advantaged) castes generally don’t allow the women of their households to work on casual jobs (or casual labour) [33,41,42].

Women in urban areas have substantially higher wages, with more than triple of the wages in rural areas both in 2004–05 and 2011–12 once again bringing to the forefront the acute rural-urban divide in India. Also, Central region followed by the Eastern have the lowest mean wages among women in India, both in rural and urban settings. And the lagging of Central and Eastern regions when it comes to economic and non-economic outcomes among Indian women is in line with existing scholarship on the subject, for example, [32,43] have similar conclusions in the context of per capita income and nutrition, respectively. At the national as well as regional level, caste-based wage disparities are profound with SC/ST women consistently earning the least, followed by the OBC women. In the religious groups, women belonging to the Muslim religion, consistently earned the least wages which is also in line with the existing narrative of condition of Muslims vis-à-vis other communities in India [35,43–45].

The overall wage inequality (based on Gini coefficient) among women in India is extremely high (around 60%) and has increased during 2004–05–2011–12. Regionally, (as expected) the Eastern and the Central regions exhibited highest inequality whereas, the region of South consistently recorded the lowest. It is worth noting here that the Southern region consists of the states which are the most advanced in terms of socio-economic-demographic outcomes [43, 10, and the references there in]. Urban areas have substantially higher wage inequality among women compared to the rural areas both in 2004–05 and 2011–12. Caste-wise, the wage inequality observed within the SC/ST and OBC women is lower than the women belonging to the “Others” (“upper”) caste category.

The main conclusions from the decomposition of total inequalities into the within and between components for various groups using MLD for 2004–05 and 2011–12 are – first, the results for overall wage inequalities based on MLD corroborate the findings using Gini coefficient (thus bringing out the robustness of this study); second, the major part of total wage inequality in all the cases is contributed by the within component which in some cases is more than 90% of the total wage inequality; third, at the all-India level, the between group wage inequality is highest (more than 20% of the total during the study period) in the case of rural/urban divide (which is not surprising given the earlier findings) followed by caste-based categorization; fourth in both the above cases (and even in the case of religion-based categorization), the between group wage inequality has reduced during the study period, clearly showing that the increase in total wage inequality during the study period is driven by the within group inequalities; fifth, same is the case with the between group (region) inequality which is high but has reduced during the study period; and last but not the least, if we observe the patterns of between group inequalities based on rural/urban, caste and religion in the various geographical regions of India, we find that the between group components are consistently relatively higher (as expected based on other earlier trends) in the Northern, Central and Eastern regions.

Though our study has strengths in terms of – using a nationally representative (and widely acceptable) data from the IHDS; using multiple (standard and acceptable) measures which add cross-checks and robustness to the analysis; and using a comprehensive and critical analysis; it also suffers from a few limitations, such as, the data is limited up to 2011–12 and changes might have happened post-2012 due to changes in socio-economic and policy environment. However, if we closely observe the debate on labour force participation and wages of women in India, we find that not much has changed during the last fifteen years or so [33,46]. Therefore, the valuable insights into wage disparities among women in India of this study, remain relevant for understanding the historical context and structural aspects of wage disparities among women in India. Also, future research can extend the findings of this study as well as add more nuanced insights on socio-economic inequality in wages among India, once new data becomes available.

Finally, the study insights underscore the need for targeted policy interventions to mitigate wage disparities and promote inclusive growth for women across different socioeconomic backgrounds. Focus needs to be on both within group as well as between group inequalities with increased attention to certain section of women. The persistent wage disparities for SC/ST, OBC, and Muslim women—particularly in socioeconomically disadvantaged regions (Central and Eastern regions of India) reflect systemic barriers. Occupational segmentation could be pushing marginalized women into informal, low-paying jobs, while disparities in education may limit their access to better opportunities. Policies like strengthening education and skill development through scholarships and vocational training, stronger labor protections, ensuring social security benefits, maternity leave, workplace safety can be aimed at. Such factors can be explored in the future research based on which effective, evidence-based solutions can be provided. Also, as suggested by [32,43,47], “one size fits all” kind of strategy might not be effective in this context and a multi-pronged flexible strategy framework focusing not only on increasing average wages but also on reducing the socioeconomic inequality in wages might be the need of the hour.

## Supporting information

Kdensity.zip Fig (a): Kernal Density Graph for Rural area, 2004–05 and 2011–12; Fig (b): Kernal Density Graph for Urban areas, 2004–05 and 2011–12. (ZIP)
