# Supplementary figures and images for: Examining wage inequality among women in India: A multidimensional analysis of socio-economic disparities
Source: PLoS One. 2025 Apr 24;20(4):e0320940. doi: 10.1371/journal.pone.0320940 (PMC12021184; doi:10.1371/journal.pone.0320940)

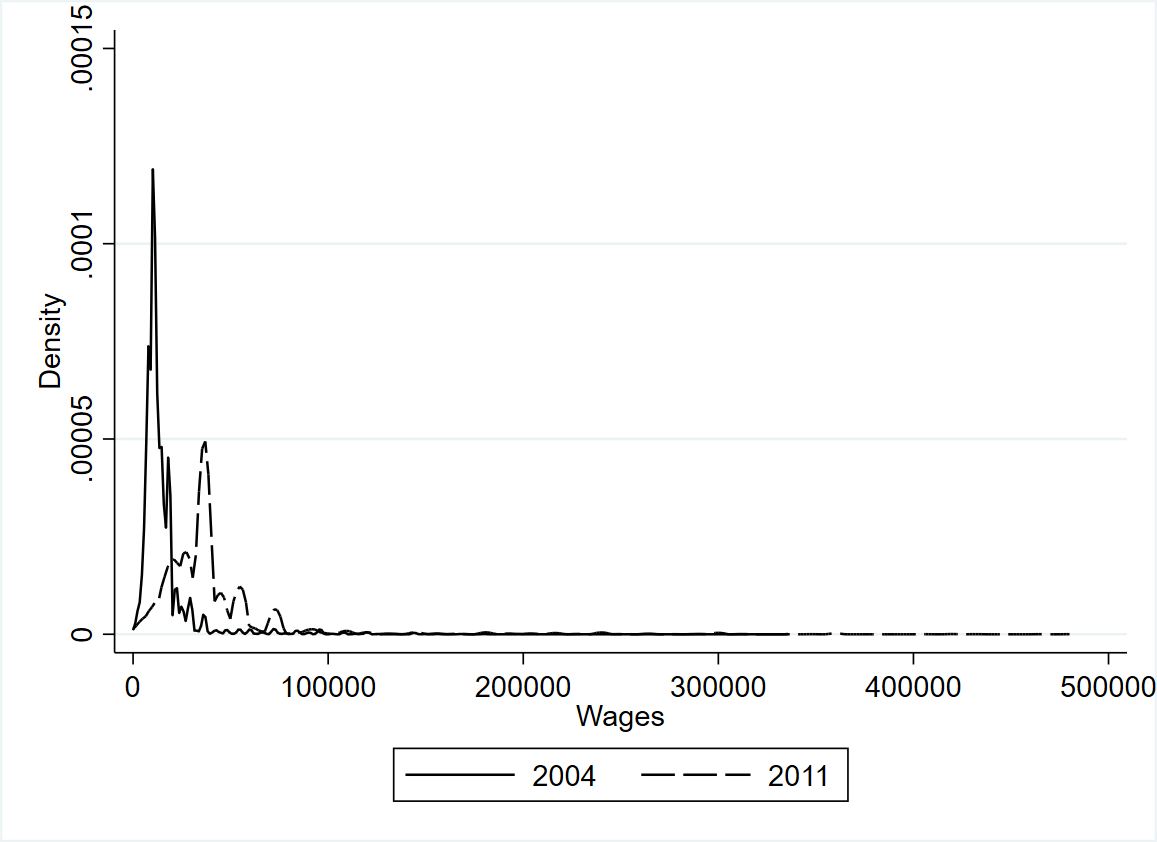

Supplement: Kdensity.zip — (ZIP) [file pone.0320940.s001.zip › Fig (a).tif]

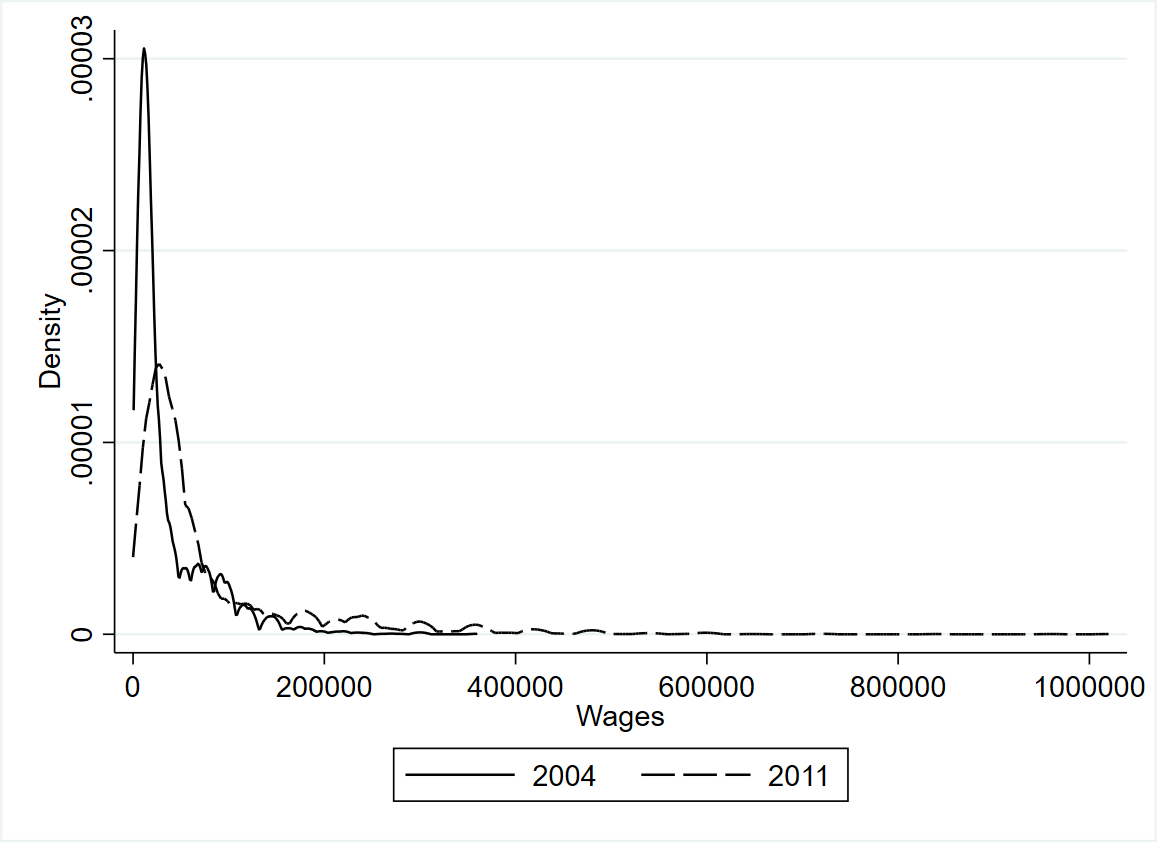

Supplement: Kdensity.zip — (ZIP) [file pone.0320940.s001.zip › Fig (b).tif]
